# Reduction of contrast medium for transcatheter aortic valve replacement planning using a spectral detector CT: a prospective clinical trial

**DOI:** 10.1007/s00330-023-10403-x

**Published:** 2023-11-18

**Authors:** Isabel L. Langenbach, Marcel C. Langenbach, Thomas Mayrhofer, Borek Foldyna, David Maintz, Konstantin Klein, Hendrik Wienemann, Kathrin B. Krug, Martin Hellmich, Matti Adam, Claas P. Naehle

**Affiliations:** 1 Cardiovascular Imaging Research Center, Department of Radiology, Massachusetts General Hospital, Harvard Medical School, 165 Cambridge Street, Suite 400, Boston, MA 02114 USA; 2 Institute for Diagnostic and Interventional Radiology, University Hospital Cologne, Cologne, Germany; 3 School of Business Studies, Stralsund University of Applied Sciences, Stralsund, Germany; 4 Clinic III for Internal Medicine, Faculty of Medicine, University of Cologne, University Hospital Cologne, Cologne, Germany; 5 Institute of Medical Statistics and Computational Biology, Medical Faculty, University of Cologne, Cologne, Germany; 6 Radiologische Allianz, Hamburg, Germany

**Keywords:** Aortic valve, Aortic valve stenosis, Tomography (X-ray computed), Transcatheter aortic valve replacement, Contrast media

## Abstract

**Introduction:**

This study investigated the use of dual-energy spectral detector computed tomography (CT) and virtual monoenergetic imaging (VMI) reconstructions in pre-interventional transcatheter aortic valve replacement (TAVR) planning. We aimed to determine the minimum required contrast medium (CM) amount to maintain diagnostic CT imaging quality for TAVR planning.

**Methods:**

In this prospective clinical trial, TAVR candidates received a standardized dual-layer spectral detector CT protocol. The CM amount (Iohexol 350 mg iodine/mL, standardized flow rate 3 mL/s) was reduced systematically after 15 patients by 10 mL, starting at 60 mL (institutional standard). We evaluated standard, and 40- and 60-keV VMI reconstructions. For image quality, we measured signal-to-noise ratio (SNR), contrast-to-noise ratio (CNR), and diameters in multiple vessel sections (i.e., aortic annulus: diameter, perimeter, area; aorta/arteries: minimal diameter). Mixed regression models (MRM), including interaction terms and clinical characteristics, were used for comparison.

**Results:**

Sixty consecutive patients (mean age, 79.4 ± 7.5 years; 28 females, 46.7%) were included. In pre-TAVR CT, the CM reduction to 40 mL is possible without affecting the image quality (MRM: SNR: –1.1, *p* = 0.726; CNR: 0.0, *p* = 0.999). VMI 40-keV reconstructions showed better results than standard reconstructions with significantly higher SNR (+ 6.04, *p* < 0.001). Reduction to 30 mL CM resulted in a significant loss of quality (MRM: SNR: –12.9, *p* < 0.001; CNR: –13.9, *p* < 0.001), regardless of the reconstruction. Across the reconstructions, we observed no differences in the metric evaluation (*p* > 0.914).

**Conclusion:**

Among TAVR candidates undergoing pre-interventional CT at a dual-layer spectral detector system, applying 40 mL CM is sufficient to maintain diagnostic image quality. VMI 40-keV reconstructions improve the vessel attenuation and are recommended for evaluation.

**Clinical relevance statement:**

Contrast medium reduction to 40 mL in pre-interventional transcatheter aortic valve replacement CT using dual-energy CT maintains image quality, while 40-keV virtual monoenergetic imaging reconstructions enhance vessel attenuation. These results offer valuable recommendations for interventional transcatheter aortic valve replacement evaluation and potentially improve nephroprotection in patients with compromised renal function.

**Key Points:**

• *Patients undergoing transcatheter aortic valve replacement (TAVR), requiring pre-interventional CT, are often multimorbid with impaired renal function.*

• *Using a spectral detector dual-layer CT, contrast medium reduction to 40 mL is feasible, maintaining diagnostic image quality.*

• *The additional application of virtual monoenergetic image reconstructions with 40 keV improves vessel attenuation significantly in clinical practice.*

**Supplementary Information:**

The online version contains supplementary material available at 10.1007/s00330-023-10403-x.

## Introduction

Aortic valve stenosis affects a significant portion of the aging population over 65 years worldwide with a poor prognosis [1]. Patients with a symptomatic disease have more than 50% mortality at two years unless an aortic valve replacement is performed promptly [2]. Transcatheter aortic valve replacement (TAVR) has become an established therapeutic procedure for patients with high-risk or contraindications for open surgery [3–5] and is increasingly performed in low- or intermediate-risk patients [6]. Considering the rapid aging of the population worldwide, the number of TAVR procedures is likely to increase in the future [7].

Pre-procedural contrast-enhanced computed tomography (CT) imaging is essential for planning the correct prosthesis size and the aortic access route. It provides comprehensive non-invasive information about the valve, aortic root dimensions, and aortic and iliac artery anatomy before TAVR. The degree of calcification and distance of the coronary ostia from the annulus plane are also essential for the optimal selection of the prosthesis model and size [8, 9].

Dual-energy or spectral computed tomography (DECT) has shown favorable results in vascular imaging because of its potential for post-processing to increase vascular contrast [10]. A central technique in DECT post-processing is virtual monoenergetic imaging (VMI), which allows for image reconstructions at a desired hypothetical energy level. Contrast-enhanced vessel attenuation increases with decreasing X-ray energies since the K-edge of iodine is closer to the lower energy spectra. VMI reconstructions at lower keV thus facilitate increased soft tissue and iodine contrast and have increased vessel attenuation at lower doses of contrast medium (CM) [11, 12].

Patients referred for TAVR are often elderly, multimorbid, and a clinically significant renal impairment is common in this population, putting them at risk for acute kidney failure after receiving intravenous iodized CM. Contrast-induced nephropathy (CIN) is a potentially severe complication associated with the application of intravenous iodized CM. To minimize this risk, using the least amount of CM as reasonably possible for diagnostic purposes is essential [13]. Signal-to-noise ratio (SNR) and contrast-to-noise ratio (CNR) are widely used as quantitative parameters of image quality and allow for improved visualization and more accurate diagnosis [10].

This study systematically investigates the reduction of iodized CM to a minimum amount needed to achieve sufficient diagnostic CT imaging data. We further hypothesize that low-keV VMI reconstructions improve the vessel attenuation compared to the standard reconstruction, thus additionally supporting a reduced amount of CM.

## Material and methods

### Patient selection and study design

The HIPAA-compliant study protocol was approved by the local institutional review board (IRB) (IRB No. 21–1245, German clinical trials register DRKS00025702) as a quality assurance trial. Written informed consent was obtained before inclusion. Patients were consecutively enrolled for this prospective study between June 2021 and November 2021. The systematic reduction of CM followed the recommendations for CM applications stated in our institutional guidelines for CT angiography, which is according to the actual Society of Cardiovascular Computed Tomography (SCCT) guidelines for TAVR imaging [14]. Inclusion criteria were clinical indication for TAVR with relevant aortic valve stenosis requiring a pre-interventional CT examination, a CT examination according to the local standard pre-TAVR CT protocol, and a scan on our dual-layer spectral detector CT (IQon, Philips Healthcare). Patients with known absolute contra-indication for CT or the application of iodized CM (e.g., allergy) were excluded.

### Contrast protocol

Our standard protocol for administering CM Iohexol 350 mg iodine/mL (Accupaque™ 350, GE Healthcare Buchler GmbH & Co. KG.) in the baseline cohort consisted of a bolus of 60 mL, followed by a 60-mL saline chaser, both at a rate of 3 mL/s. After evaluating 15 patients, the protocol was adjusted. We reduced the CM bolus in steps of 10 mL and increased the saline chaser by 10 mL to maintain a stable total volume of 120 mL with a fixed CM injection rate of 3 mL/s. Subsequent reductions were made to 50 mL CM and 70 mL saline chaser, then 40 mL CM and 80 mL saline chaser, and at least 30 mL CM and 90 mL saline chaser. The injection rate was not modulated during the application or changed with the reduction of the CM amount to achieve optimal comparability between all groups and reduce potential confounding effects. The CM was typically administered via the right cubital vein using an automatic injection system (Medrad® Stellent Injection System, Medrad, Bayer), with contrast bolus tracking set to a threshold of 120 HU in the ascending aorta and a threshold delay of 4.9 s.

### Dual-energy computed tomography data acquisition and image reconstruction

CT datasets were acquired with a dual-layer spectral detector CT using retrospective ECG-gating acquisition mode, extending from the clavicles to the femoral heads. Scan parameters were as follows: slice thickness of 2 mm, tube voltage 120 kV, tube current time product 130 mAs, 64 × 0.625 mm collimation, 0.33 s rotation time, pitch of 0.3. The CT scan duration of the CT ECG-gated angiography was noted. The images were post-processed using a conventional statistical iterative reconstruction algorithm (IMR level 2, Philips Healthcare) and a standard cardiac kernel. For each patient, VMI reconstructions were calculated for the contrast-enhanced images in two different keV levels (VMI 40 and 60 keV) on a dedicated image processing console (IntelliSpace Portal, Philips Healthcare) using the same iterative reconstruction level and reconstruction kernel as the standard dataset. Reconstructions were created immediately following the examination at the scanner and transferred to the local picture archiving and communication system (PACS).

### Quantitative image analysis

For quantitative assessment, we measured the maximum and minimum diameters, perimeter, and area of the aortic annulus, and minimum vessel diameters of the ascending, descending, and abdominal aorta, as well as bilateral common iliac, external iliac, and common femoral artery runoffs.

Furthermore, the study aimed to determine the image quality in various regions of the arterial vessels through all reconstructions. For a quantitative assessment, regions of interest (ROIs) were placed centrally in the vessel of the ascending aorta, aortic arch, descending thoracic aorta, descending abdominal aorta, common iliac arteries on both sides, external iliac arteries on both sides, and common femoral arteries on both sides. The ROI size was chosen individually for each patient, ensuring the largest size possible while preserving the vessel wall. The CT images were automatically linked to ensure comparability among all reconstructions, and the ROIs were projected in each series to guarantee the correct position and size. Signal attenuations in mean HU values and image noise, defined as standard deviation (SD), were measured at a multimodal workstation. Additionally, measurements were made in the subcutaneous adipose tissue of the lower abdomen to calculate image contrast. Standardized ROIs with a diameter of 10 mm were used for these measurements. These measurements were performed in triplicate and then averaged to minimize measurement inaccuracies. SNR and CNR as standard measures of image quality and vessel attenuation were calculated following the study of Willemink et al [15].

### Qualitative image analysis

Two independent readers (M.L., K.K.) with experience in cardiovascular imaging (equivalent SCCT level of accreditation 2) performed the qualitative image analysis using a PACS (Dedalus Healthcare) imaging workstation. A 5-point Likert scale was employed to individually assess the image quality of all contrast medium groups and reconstruction: 1, non-diagnostic; 2, diagnostic despite impairment by image noise and/or low or significantly inhomogeneous intraluminal contrast opacification; 3, moderate image noise with sufficient vessel attenuation, intraluminal contrast opacification may be present; 4, good vessel contrast and attenuation, low image noise; 5, excellent, no diagnostic limitations. The readers visually evaluated the diagnostic capability of the CT for TAVR planning, focusing specifically on the pertinent anatomical structures with individual evaluation of multiple vessel segments (aortic valve/annulus, ascending, descending, and abdominal aorta, bilateral common iliac, external iliac, and common femoral artery runoffs).

### Statistical evaluation

Data was collected in a computerized database (Excel®, Microsoft Corp.). Quantitative variables were described with mean and SD or median, minimum, and maximum qualitative variables with absolute and relative frequency (%). Categorical variables were compared using the Fisher exact test, and continuous variables were compared using the one-way ANOVA test for normal distribution or the Kruskal–Wallis *t*-test for asymmetric distribution. We used multilevel mixed-effects linear regression models to assess the influence of the CM amount and VMI reconstructions, including interaction terms and clinical characteristics (age, sex, BMI, Agatston score, atrial fibrillation, and left ventricular ejection fraction) and controlling for possible autocorrelation due to multiple regions per patient. The interrater reliability was calculated using cross tables and determining Cohen’s Kappa, evaluated according to Landis/Koch [16]. In the essentially exploratory analysis, *p* values ≤ 0.05 were considered statistically significant. Statistical analyses were performed using Stata (Stata 17, Stata Corp.). The sample size for each group in this study was determined by considering the existing literature, anticipating a significant difference of 0.75 standard deviations [10]. To ensure a statistical power of 0.80 and account for multiple comparisons using the Bonferroni correction, a sample size of *n* = 12 per group was estimated. Factoring in an expected dropout rate of 20%, the final calculated group size was* n* = 15.

## Results

### Patient cohort

**Fig. 1:**
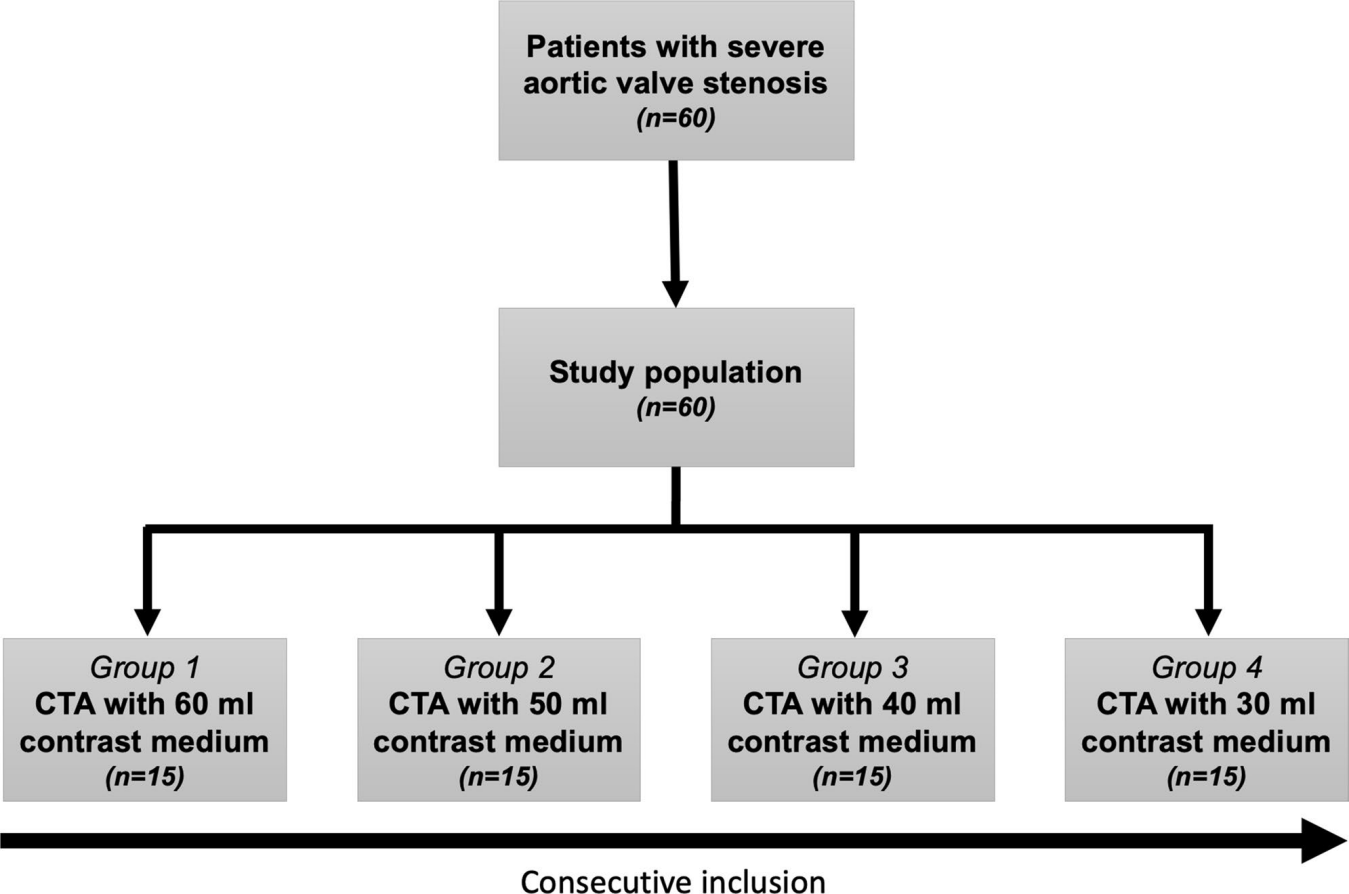
Flowchart of study enrollment. *CTA*, computed tomography angiography

A total of 60 consecutive patients (79.4 ± 7.5 years; range, 54–97 years), including 28 (46.7%) female patients, were prospectively enrolled in this study (flowchart, Fig. 1). All CT examinations were performed without complications. Thus, no examinations had to be excluded from this study. We observed no differences regarding clinical characteristics between all 4 CM groups (*p* ≥ 0.271). The patient’s mean body mass index (BMI) was 26.2 ± 5.0 kg/m^2^, ranging from 16.9 to 40.5 kg/m^2^. Nearly one-third of the patients (31.7%) suffered from chronic renal failure. A detailed overview of the individual characteristics of the investigated patient cohort is provided in Table 1.

**Table 1:** Baseline characteristics, overall, and comparing the different contrast medium groups

mean ± SD, median (Q1–Q3), or *N* (%)	Overall (*N* = 60)	Group 1 60 mL (*N* = 15)	Group 2 50 mL (*N* = 15)	Group 3 40 mL (*N* = 15)	Group 4 30 mL (*N* = 15)	*p* value
Age, years	79.4 ± 7.5	79.0 ± 7.3	77.4 ± 10.1	80.0 ± 7.5	81.3 ± 4.4	0.554
Sex						0.583
Male	32 (53.3)	8 (53.3)	6 (40.0)	10 (66.7)	8 (53.3)	
Female	28 (46.7)	7 (46.7)	9 (60.0)	5 (33.3)	7 (46.7)	
BMI, kg/m^2^	26.2 ± 5.0	24.9 ± 4.6	26.3 ± 6.2	25.5 ± 3.5	28.1 ± 5.2	0.326
Agatston Score	2,528.0 [1,724.3–4,109.8]	2,227.0 [1,445.5–4,244.0]	2,363.0 [1,301.0–3,216.5]	3,628.0 [2,304.0–4,708.0]	2,415.0 [1,897.0–2,811.5]	0.222
CTA scan duration, s	17.2 ± 2.1	17.3 ± 2.1	16.7 ± 2.2	17.4 ± 2.2	17.3 ± 2.2	0.831
EuroScore II	1.8 [0.9–3.6]	2.6 [1.2–3.6]	2.4 [0.6–4.1]	1.6 [0.8–2.5]	1.7 [0.8–2.7]	0.621
Creatinine, mg/dL	1.0 [0.8–1.2]	0.9 [0.8–1.1]	1.0 [0.8–1.5]	1.0 [0.8–1.1]	1.1 [0.8–1.3]	0.740
GFR, mL/min/1.73 m^2^	61.4 ± 26.2	59.0 ± 25.8	63.7 ± 36.1	68.3 ± 19.6	54.7 ± 20.5	0.532
Chronic renal failure						0.611
No	41 (68.3)	11 (73.3)	9 (60.0)	12 (80.0)	9 (60.0)	
Yes	19 (31.7)	4 (26.7)	6 (40.0)	3 (20.0)	6 (40.0)	
EF						0.979
Normal (> 50%)	46 (76.7)	11 (73.3)	12 (80.0)	12 (80.0)	11 (73.3)	
Moderately reduced (41–50%)	7 (11.7)	2 (13.3)	2 (13.3)	2 (13.3)	1 (6.7)	
Reduced (31–40%)	4 (6.7)	1 (6.7)	1 (6.7)	0 (0.0)	2 (13.3)	
Severely reduced (< 30%)	3 (5.0)	1 (6.7)	0 (0.0)	1 (6.7)	1 (6.7)	

Categorical variables were compared using the Fisher exact test, continuous variables using the one-way ANOVA test for normal distribution, or the Kruskal–Wallis *t*-test for asymmetric distribution

*BMI*, body mass index; *CTA*, computed tomography angiography; *GFR*, glomerular filtration rate; *EF*, ejection fraction

After the clinical and radiological TAVR evaluation, 46 patients (76.7%) from the cohort received a TAVR. Of the 14 patients (23.3%) who did not undergo a TAVR procedure, nine patients (15.0%) received a surgical valve replacement.

### Quantitative image analysis

Comparing the metric measurements of the aortic annulus, the aorta, and pelvic arteries facilitating the different image reconstructions, no significant differences between the standard, and the VMI 40 keV and 60 keV could be observed (*p* ≥ 0.914) (Table 2).

**Table 2:** Comparison of the quantitative metric measurements in the different vessel sections using standard, and VMI 40-keV and VMI 60-keV reconstructions

Vessel section (mean ± SD)	Standard (*N* = 60)	VMI 40 keV (*N* = 60)	VMI 60 keV (*N* = 60)	*p* value
AoAnulus diameter (max.)	33.3 ± 4.5	33.2 ± 4.4	33.3 ± 4.5	0.995
AoAnulus diameter (min.)	29.0 ± 4.0	29.3 ± 3.9	29.3 ± 4.1	0.914
AoAnulus perimeter	101.2 ± 13.5	101.3 ± 13.3	101.3 ± 13.5	0.999
AoAnulus area	773.6 ± 202.8	779.1 ± 200.3	778.8 ± 202.8	0.986
AoAsc	35.6 ± 4.6	35.6 ± 4.5	35.6 ± 4.5	0.999
AoArc	27.2 ± 3.5	27.2 ± 3.5	27.2 ± 3.5	1.000
ThoAo	25.2 ± 3.3	25.2 ± 3.2	25.0 ± 3.2	0.998
AbdAo	18.7 ± 2.9	18.7 ± 2.8	18.7 ± 2.8	1.000
RCIA	10.5 ± 2.1	10.5 ± 2.1	10.5 ± 2.1	0.995
LCIA	10.6 ± 2.2	10.6 ± 2.2	10.6 ± 2.2	0.999
REIA	8.1 ± 1.7	8.1 ± 1.7	8.1 ± 1.7	0.998
LEIA	8.1 ± 1.5	8.0 ± 1.5	8.1 ± 1.5	0.990
RCFA	8.3 ± 1.6	8.3 ± 1.6	8.3 ± 1.6	0.998
LCFA	8.3 ± 1.5	8.3 ± 1.5	8.3 ± 1.4	1.000

Aortic annulus area in mm^2^, all other values in mm

*AoAnulus*, aortic annulus; *AoAsc*, ascending aorta; *AoArc*, aortic arch; *ThoAo*, thoracic descending aorta; *AbdAo*, abdominal descending aorta; *RCIA*, right common iliac artery; *LCIA*, left common iliac artery; *REIA*, right external iliac artery; *LEIA*, left external iliac artery; *RCFA*, right common femoral artery; *LCFA*, left common femoral artery

Considering overall SNR and CNR as markers for the image quality and after adjustment for all interaction terms and clinical characteristics, a significant decrease was seen for the 30 mL CM group (SNR: –12.92, *p* < 0.001; CNR: –13.86, *p* < 0.001). No significant decrease in image quality was observed for the 50 mL and 40 mL CM groups. Details are provided in Table 3. A complete overview of all interaction terms can be found in the supplementary information (S1).

**Table 3:** Mixed regression model corrected for all interaction terms (contrast medium, reconstructions, vessel area; details in S1) and clinical characteristics. Significant *p* values are indicated in bold

	SNR			CNR		
	Coef	95% Conf. Interval	*p* value	Coef	95% Conf. Interval	*p* value
Contrast medium						
60 mL	Ref			Ref		
50 mL	− 0.21	− 6.24–5.82	0.946	− 0.27	− 6.17 to 5.64	0.930
40 mL	− 1.10	− 7.23–5.04	0.726	0.00	− 6.04 to 6.04	0.999
30 mL	− 12.92	− 19.04 to − 6.81	** < 0.001**	− 13.86	− 19.87 to − 7.85	** < 0.001**
Reconstructions						
Standard	Ref			Ref		
VMI 40 keV	6.04	3.66–8.42	** < 0.001**	0.71	− 1.42 to 2.84	0.513
VMI 60 keV	− 9.80	− 12.19 to − 7.42	** < 0.001**	− 18.43	− 20.56 to − 16.30	** < 0.001**
Clinical characteristics						
Age	0.23	0.03–0.44	**0.027**	0.16	− 0.07 to 0.38	0.174
Sex *(female)*	2.92	− 0.19 to 6.03	0.066	3.20	− 0.17 to 6.57	0.063
BMI	− 0.88	− 1.18 to − 0.57	** < 0.001**	− 0.83	− 1.17 to − 0.50	** < 0.001**
Agatston Score	0.00	− 0.00 to 0.00	0.423	0.00	− 0.00 to 0.00	0.704
Atrial fibrillation	2.37	− 0.80 to 5.55	0.143	3.40	− 0.05 to 6.84	0.053
Ejection fraction *(normal (*> *50%))*	Ref					
Moderately reduced (41–50%)	− 0.73	− 5.34 to 3.89	0.758	− 2.33	− 7.34 to 2.68	0.361
Reduced (31–40%)	− 2.89	− 8.93 to 3.15	0.348	− 4.17	− 10.72 to 2.39	0.213
Severely reduced (< 30%)	5.04	− 1.70 to 11.78	0.142	6.32	− 0.99 to 13.63	0.090

*SNR*, signal-to-noise ratio; *CNR*, contrast-to-noise ratio; *BMI*, body mass index

**Fig. 2:**
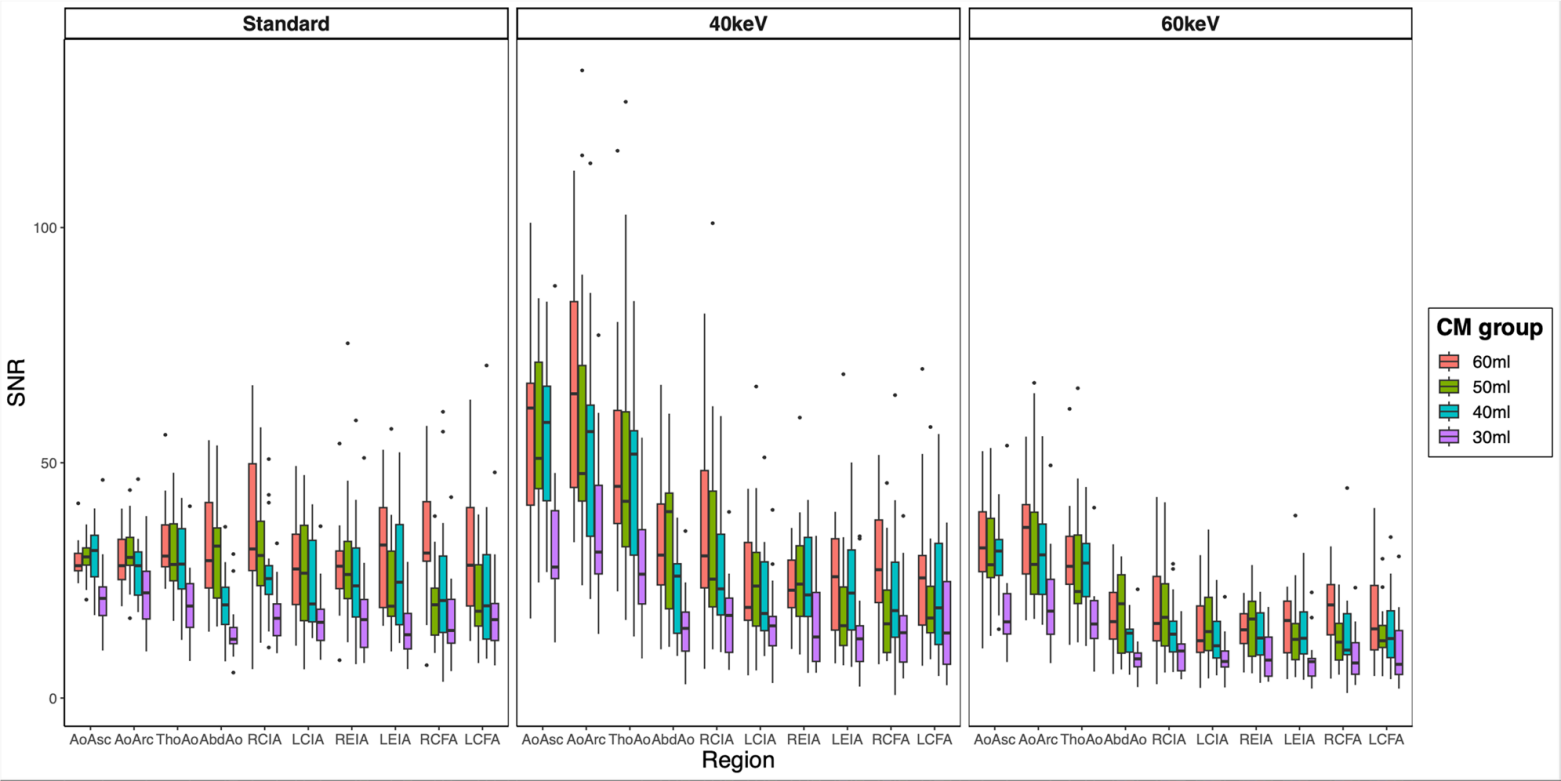
Boxplot graphic illustrating the different levels of signal-to-noise ratios in the different vessel sections, displayed for the different reconstructions (standard, VMI 40 keV, VMI 60 keV) and the different contrast medium groups. *CM*, contrast medium; *SNR*, signal-to-noise ratio; *AoAsc*, ascending aorta; *AoArc*, aortic arch; *ThoAo*, thoracic descending aorta; *AbdAo*, abdominal descending aorta; *RCIA*, right common iliac artery; *LCIA*, left common iliac artery; *REIA*, right external iliac artery; *LEIA*, left external iliac artery; *RCFA*, right common femoral artery; *LCFA*, left common femoral artery

**Fig. 3:**
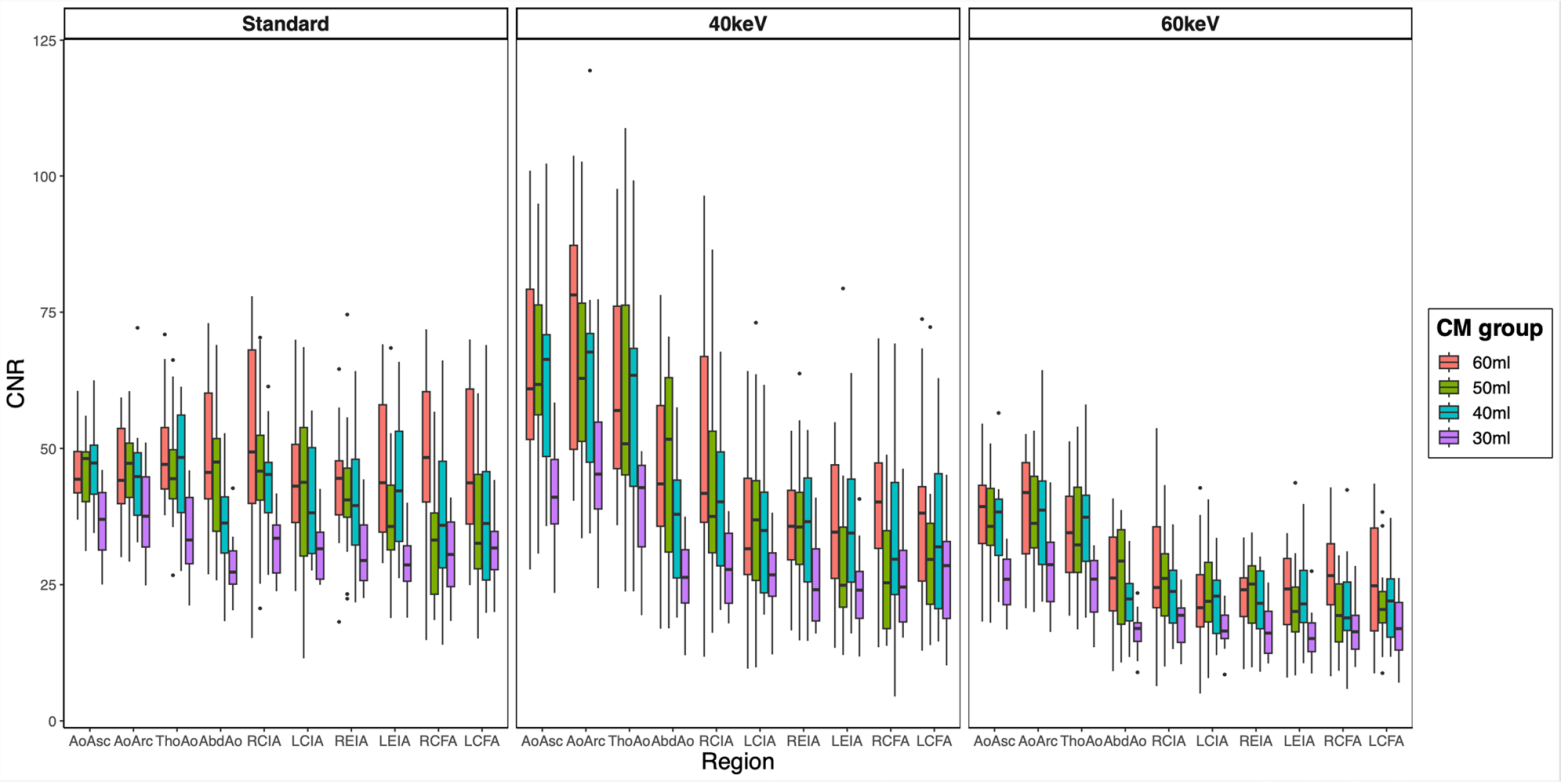
Boxplot graphic illustrating the different levels of contrast-to-noise ratios in the different vessel sections, displayed for the different reconstructions (standard, VMI 40 keV, VMI 60 keV) and the different contrast medium groups. *CM*, contrast medium; *CNR*, contrast-to-noise ratio; *AoAsc*, ascending aorta; *AoArc*, aortic arch; *ThoAo*, thoracic descending aorta; *AbdAo*, abdominal descending aorta; *RCIA*, right common iliac artery; *LCIA*, left common iliac artery; *REIA*, right external iliac artery; *LEIA*, left external iliac artery; *RCFA*, right common femoral artery; *LCFA*, left common femoral artery

Regarding the different VMI reconstructions, compared to the standard reconstruction, the VMI 40-keV reconstruction shows a significant benefit regarding the SNR (+ 6.04, *p* < 0.001) with no difference regarding the CNR (*p* = 0.513). The VMI 60-keV reconstruction was inferior to the standard regarding SNR (− 9.80, *p* < 0.001) and CNR (− 18.43,* p* < 0.001) (Table 3, Figs. 2 and 3, S2).

We further tested various clinical characteristics for their association with image quality and vessel attenuation. After adjustment for CM amount and reconstructions, BMI remains the only patient individual characteristic significantly negatively associated with the image quality (SNR: − 0.88, *p* < 0.001; CNR: − 0.83, *p* < 0.001). Age was significantly associated with the SNR (0.23, *p* = 0.027), while no significance was found regarding CNR (*p* = 0.174).

### Qualitative image analysis

**Fig. 4:**
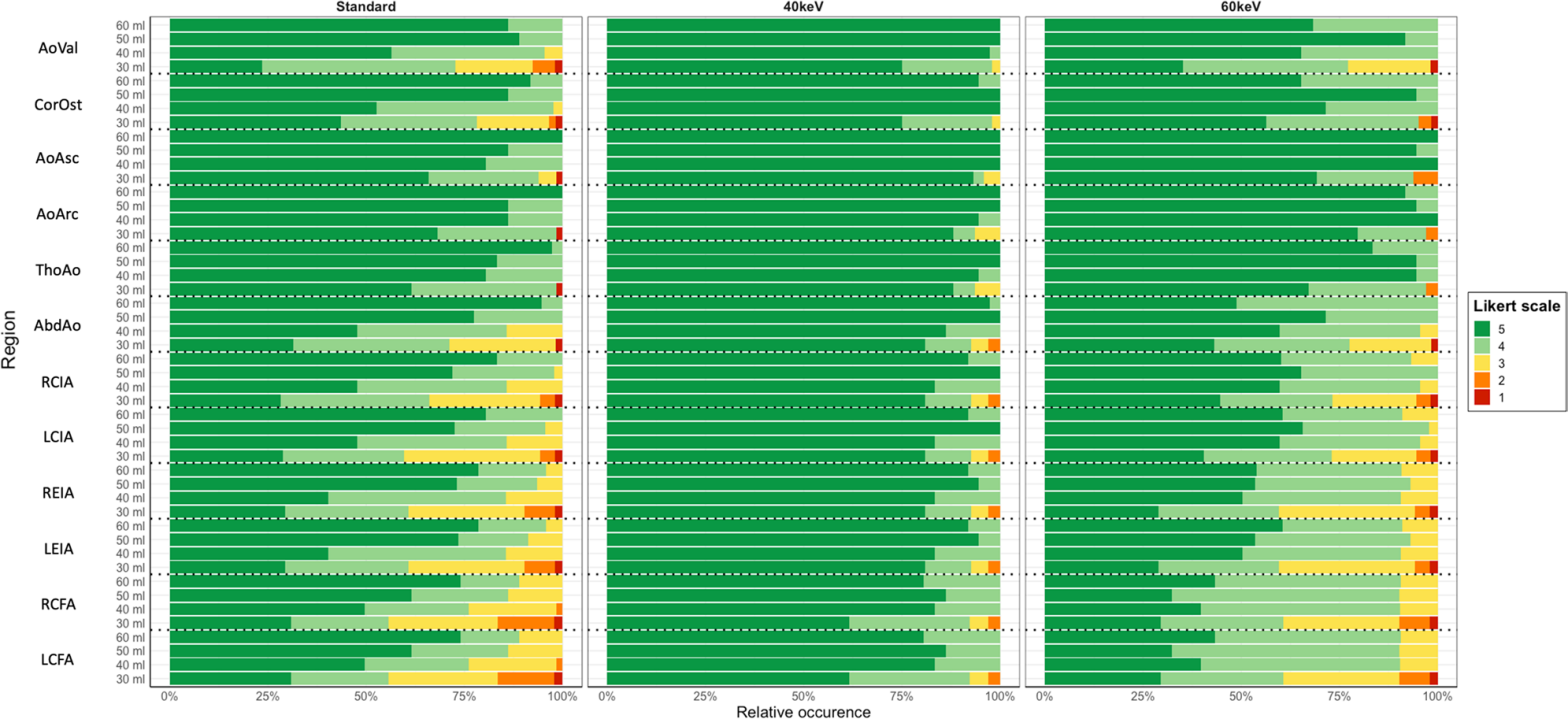
Stacked box model illustrating the qualitative analysis. Proportions of the rating based on a 5-point Likert scale for the evaluated vessel sections are displayed. *AoVal*, aortic valve; *CorOst*, coronary ostia; *AoAsc*, ascending aorta; *AoArc*, aortic arch; *ThoAo*, thoracic descending aorta; *AbdAo*, abdominal descending aorta; *RCIA*, right common iliac artery; *LCIA*, left common iliac artery; *REIA*, right external iliac artery; *LEIA*, left external iliac artery; *RCFA*, right common femoral artery; *LCFA*, left common femoral artery

**Fig. 5:**
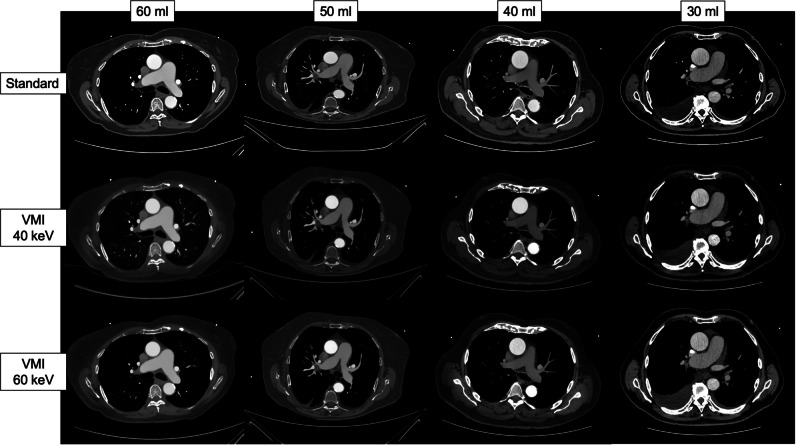
Typical CT imaging examples at the level of the ascending aorta in the different contrast medium groups in three different reconstructions (standard, VMI 40 keV, VMI 60 keV). *VMI*, virtual monoenergetic imaging

When considering the qualitative analysis using the visual evaluation based on the questionnaires, the interrater reliability of both readers showed a substantial to almost perfect agreement for the different vessel sections (*κ* = 0.763–0.923). The average image quality was rated with 5 (IQR 4–5). For the different contrast protocols and image reconstructions, the rating was 5 (IQR 5–5) (standard reconstruction)/5 (IQR 5–5) (VMI 40 keV)/5 (IQR 4–5) (VMI 60 keV) for the 60 mL group, 5 (IQR 4–5)/5 (IQR 5–5)/ 5 (IQR 4–5) for 50 mL, 4.5 (IQR 4–5)/ 5 (IQR 5–5)/5 (IQR 4–5) for 40 mL, and 4 (IQR 3–4)/5 (IQR 4–5)/ 4 (IQR 3–5) for 30 mL (Fig. 4). Comparing the different contrast protocols, in patients with the application of 30 mL CM, the image quality was rated significantly lower compared to the standard of 60 mL (− 0.7, *p* < 0.001) in the fully adjusted models. No significant difference in image quality was observed for using 50 mL (*p* = 0.904) or 40 mL (*p* = 0.396). Regarding the VMI reconstructions, using VMI 40 keV significantly increased the subjective image quality by 0.5 (*p* < 0.001) after full adjustment. The VMI 60 keV showed no significant impact based on the qualitative evaluation (*p* = 0.706). Typical imaging examples are displayed in Fig. 5.

## Discussion

This study evaluated the possible reduction of CM below the standard amount of 60 mL, ensuring diagnostic image quality for TAVR planning CT. The CM amount necessary for a sufficient TAVR planning CT can be reduced to 40 mL, providing equivalent results in image quality, vessel attenuation, and evaluability compared to the standard amount. VMI reconstructions at 40 keV improve the CT vessel attenuation based on SNR and qualitative image analysis compared with the standard reconstruction.

For TAVR patients, CM administration is mandatory for the pre-interventional CT, the invasive cardiac catheterization to exclude relevant CAD, and the TAVR procedure itself. Patients with high-grade, relevant aortic valve stenosis are primarily multimorbid and often present with impaired renal function [17]. To avoid CM-induced nephropathy, the necessarily administered CM amount for the pre-TAVR CT and the subsequent interventions should be reduced as far as diagnostically acceptable. Therefore, nephroprotection by decreasing the applied CM amount seems warranted. An analysis of the collective from the PARTNER trial showed that preoperative severe renal dysfunction (eGFR ≤ 30 mL/min) was associated with significantly increased 1-year mortality (34.4% vs. 21.5%, moderate dysfunction, vs. 20.8%, no/mild dysfunction) [18]. In addition, CT allows, at least in a large proportion of patients, evaluation of the coronary arteries with the aid of functional CT-based analysis of coronary stenoses [19, 20]. Thus, the diagnostic cardiac catheterization examination previously required before TAVR implantation could potentially be omitted in the future, leading to additional savings in CM.

One of the first studies evaluating DECT for TAVR planning was the SPECTACULAR trial by Cavallo et al. In this study, 116 patients were prospectively examined using 50 mL CM. Standard and VMI 40-keV reconstructions were compared regarding image quality and metric evaluation. The authors showed that TAVR evaluation was possible with 50 mL CM with better results using the VMI 40-keV dataset [10]. Furthermore, iodine contrast, and therefore vessel attenuation, is most pronounced at 40 keV, allowing adequate assessment of vessels even at low CM levels. Große Hokamp et al have shown this for the pulmonary arteries, based on subjective and objective CT image quality. VMI 40 keV significantly improved the vessel attenuation in this study [21]. Shuman et al showed that using VMI reconstructions at 50 keV allows adequate diagnosis of the aorta with a 50% reduction of the CM amount [22].

These results are partially in accordance with our results. We confirmed that 50 mL CM is also suitable for sufficient pre-TAVR imaging, and a CM amount reduction using VMI 40-keV reconstruction is also possible without a significant loss of image quality. Nevertheless, the benefit of the VMI 40-keV reconstruction over the standard reconstruction was only demonstrable regarding SNR but not CNR. The readers preferred the VMI 40-keV reconstruction over the standard reconstruction in the qualitative evaluation.

To the best of our knowledge, our study is the first trial to address systematically, in a comparative manner with the previous standard of 60 mL CM, the CM amount required for adequate pre-TAVR imaging using DECT data.

We further investigated the influence of clinical characteristics on image quality. With increasing BMI, the image quality decreased significantly [23]. Based on our results, the image quality with 40 mL CM was sufficient for all patients regardless of the BMI. For a further reduction of CM below 40 mL or imaging in adipose patients, consideration of BMI might be favorable. Nevertheless, a body weight–adapted CM application might be a solution to avoid non-diagnostic image quality, not only in pre-TAVR imaging. Interestingly, the patient’s age was slightly positively associated with the SNR. This might be a cofounding with other, non-investigated clinical characteristics. The underlying reason remains unclear and requires further investigation.

Comparing the individual readers revealed no significant differences for the qualitative evaluation, showing how reliable and generally well evaluable pre-TAVR CT imaging is. In particular, for the appropriate analysis of the aortic valve, the qualitative analysis of the VMI 40-keV reconstructions showed homogeneously good results, even in the groups with reduced amounts of CM, except 30 mL CM. This suggests that the reviewers accept these reconstructions well and that an evaluation is possible in many cases, even with reduced CM quantity and potentially increased image noise.

We followed the SCCT scan parameter recommendation, adapted to the available CT system, and used a protocol that allowed the acquisition of the entire examination volume with only one ECG-triggered scan and one CM application [24]. Our chosen protocol made a significant CM reduction possible even with a single craniocaudal scan. Further studies regarding the optimal application protocol with 40 mL or even 30 mL CM are necessary to achieve further progress. One potential approach to further enhance the arterial attenuation more effectively could involve utilizing a split bolus or adjusting the CM injection rate throughout the scan. Kok et al exhibited results comparable to our study by modulating the injection rate and reducing CM volume for TAVR imaging. Their study compared 53 mL CM at an injection rate of 4 mL/s with 40 mL CM at 3 mL/s [23]. Higashigaito et al employed injection rate modulation while simultaneously reducing CM volume to uphold consistent injection times for patients undergoing thoracoabdominal CT angiography [25]. Both approaches should be considered for further investigations of low CM-dose protocols for vascular or TAVR CT imaging.

Using a dual-layer or photon-counting detector CT, reconstruction of monoenergetic datasets is possible retrospectively from every acquired CT scan. A dedicated dual-energy acquisition protocol is not required. This is the crucial difference from other dual-energy approaches for which a dual-energy acquisition protocol must be defined in advance. Other studies, such as those by Grant et al and Albrecht et al, have shown that CT scanners using other dual-source techniques can be similarly employed for CM reduction [26–28]. The disadvantage of the dual-layer spectral technology is a slower rotation speed, especially compared to dual-source systems, and the resulting increased radiation dose. However, in the TAVR population with primarily elderly patients, this is not as significant as the achieved CM reduction [29].

The present study has several limitations. Being a single-center study, a multicenter and a multi-vendor follow-up trial would be desirable to generalize the results. The number of patients included and the small cohort size due to the pilot character of the study are other important limitations. However, due to the significant results obtained, the systematic analysis performed on the reduction of the CM amount now allows further studies for a more precise evaluation of the CM protocol and for the analysis of influencing factors to determine the necessary amount of CM for individual patients, if required. The primary aim of this study was to define a generalizable CM protocol valid for all patients. The administration of CM in the study was performed according to the valid standard operating procedure (SOP) in our institute, independent of body weight. Body weight–adapted CM application is possibly superior to fixed CM administration in TAVR patients, requiring further investigation. Finally, no analysis of the renal function after the CT and interventions was performed as this was not part of the study aims. It is of note that renal function in the context of TAVR depends not only on the CM administered during pre-interventional CT but also on various other factors, such as the amount of CM administered during TAVR, and postinterventional course, among others. Therefore, a much larger cohort is necessary to show the impact of the CM applied during CT on renal function.

This prospective study demonstrated that reducing the CM amount used in CT imaging for TAVR planning on dual-layer spectral detector CT to 40 mL is possible without compromising the diagnostic image quality. VMI 40 keV reconstructions showed better results regarding SNR and in the qualitative analysis and should be preferred for image evaluation. Reduction to 30 mL CM shows a significant loss of image quality and is therefore not recommended for clinical practice. These results offer valuable recommendations for TAVR evaluation and can potentially improve nephroprotection in this fragile patient cohort with often compromised renal function.

### Supplementary Information

Below is the link to the electronic supplementary material.

Supplementary file1 (DOCX 20 KB)

## Abbreviations

BMI: Body mass index
CIN: Contrast-induced nephropathy
CM: Contrast medium
CNR: Contrast-to-noise ratio
CT: Computed tomography
DECT: Dual-energy computed tomography
ROI: Regions of interest
SNR: Signal-to-noise ratio
TAVR: Transcatheter aortic valve replacement
VMI: Virtual monoenergetic imaging

## Declarations

### Guarantor

The scientific guarantor of this publication is Marcel C. Langenbach, MD.

### Statistics and biometry

Thomas Mayrhofer and Martin Hellmich kindly provided statistical advice for the manuscript.

### Study subjects or cohorts overlap

No study subjects have been previously reported.

### Methodology

• prospective

• experimental Study

• performed at one institution

## References

[1] Maganti K, Rigolin VH, Sarano ME, Bonow RO (2010). Valvular heart disease: diagnosis and management. Mayo Clin Proc.

[2] Zajarias A, Cribier AG (2009). Outcomes and safety of percutaneous aortic valve replacement. J Am Coll Cardiol.

[3] Smith CR, Leon MB, Mack MJ (2011). Transcatheter versus surgical aortic-valve replacement in high-risk patients. N Engl J Med.

[4] Kodali SK, Williams MR, Smith CR (2012). Two-year outcomes after transcatheter or surgical aortic-valve replacement. N Engl J Med.

[5] Holmes DR, Mack MJ (2011). Transcatheter valve therapy: a professional society overview from the American College of Cardiology Foundation and the Society of Thoracic Surgeons. Ann Thorac Surg.

[6] Leon MB, Smith CR, Mack MJ (2016). Transcatheter or surgical aortic-valve replacement in intermediate-risk patients. N Engl J Med.

[7] Blumenstein J, Kim WK, Liebetrau C (2015). Challenges of coronary angiography and intervention in patients previously treated by TAVI. Clin Res Cardiol.

[8] Kasel AM, Cassese S, Bleiziffer S (2013). Standardized imaging for aortic annular sizing: implications for transcatheter valve selection. JACC Cardiovasc Imaging.

[9] Rossebø AB, Pedersen TR, Boman K (2008). Intensive lipid lowering with simvastatin and ezetimibe in aortic stenosis. N Engl J Med.

[10] Cavallo AU, Patterson AJ, Thomas R (2020). Low dose contrast CT for transcatheter aortic valve replacement assessment: results from the prospective SPECTACULAR study (spectral CT assessment prior to TAVR). J Cardiovasc Comput Tomogr.

[11] Martin SS, Albrecht MH, Wichmann JL (2017). Value of a noise-optimized virtual monoenergetic reconstruction technique in dual-energy CT for planning of transcatheter aortic valve replacement. Eur Radiol.

[12] Jhaveri KD, Saratzis AN, Wanchoo R, Sarafidis PA (2017). Endovascular aneurysm repair (EVAR)- and transcatheter aortic valve replacement (TAVR)-associated acute kidney injury. Kidney Int.

[13] Rudnick MR, Leonberg-Yoo AK, Litt HI, Cohen RM, Hilton S, Reese PP (2020). The controversy of contrast-induced nephropathy with intravenous contrast: what is the risk?. Am J Kidney Dis.

[14] Gulati M, Levy PD, Mukherjee D (2021). 2021 AHA/ACC/ASE/CHEST/SAEM/SCCT/SCMR Guideline for the Evaluation and Diagnosis of Chest Pain: a report of the American College of Cardiology/American Heart Association Joint Committee on Clinical Practice Guidelines. Circulation.

[15] Willemink MJ, Leiner T, de Jong PA (2013). Iterative reconstruction techniques for computed tomography part 2: initial results in dose reduction and image quality. Eur Radiol.

[16] Landis JR, Koch GG (1977). The measurement of observer agreement for categorical data. Biometrics.

[17] Bittner DO, Arnold M, Klinghammer L (2016). Contrast volume reduction using third generation dual source computed tomography for the evaluation of patients prior to transcatheter aortic valve implantation. Eur Radiol.

[18] Thourani VH, Forcillo J, Beohar N (2016). Impact of preoperative chronic kidney disease in 2,531 high-risk and inoperable patients undergoing transcatheter aortic valve replacement in the PARTNER Trial. Ann Thorac Surg.

[19] Wienemann H, Langenbach MC, Mauri V et al (2022) Feasibility and comparison of resting full-cycle ratio and computed tomography fractional flow reserve in patients with severe aortic valve stenosis. J Cardiovasc Dev Dis 9 10.3390/jcdd9040116 PMC9030550 35448092

[20] Gohmann RF, Pawelka K, Seitz P (2022). Combined cCTA and TAVR planning for ruling out significant CAD: added value of ML-based CT-FFR. JACC Cardiovasc Imaging.

[21] Große Hokamp N, Kessner R, Van Hedent S, Graner FP, Gupta A, Gilkeson R (2018). Spectral detector computed tomography pulmonary angiography: improved diagnostic assessment and automated estimation of window settings angiography of pulmonary arteries from novel spectral detector computed tomography provides improved image quality if settings are adjusted. J Comput Assist Tomogr.

[22] Shuman WP, Chan KT, Busey JM, Mitsumori LM, Koprowicz KM (2016). Dual-energy CT aortography with 50% reduced iodine dose versus single-energy CT aortography with standard iodine dose. Acad Radiol.

[23] Kok M, Turek J, Mihl C (2016). Low contrast media volume in pre-TAVI CT examinations. Eur Radiol.

[24] Blanke P, Weir-McCall JR, Achenbach S (2019). Computed tomography imaging in the context of transcatheter aortic valve implantation (TAVI)/transcatheter aortic valve replacement (TAVR): an expert consensus document of the Society of Cardiovascular Computed Tomography. JACC Cardiovasc Imaging.

[25] Higashigaito K, Mergen V, Eberhard M (2023). CT angiography of the aorta using photon-counting detector CT with reduced contrast media volume. Radiol Cardiothorac Imaging.

[26] Albrecht MH, Scholtz JE, Kraft J (2015). Assessment of an advanced monoenergetic reconstruction technique in dual-energy computed tomography of head and neck cancer. Eur Radiol.

[27] Grant KL, Flohr TG, Krauss B, Sedlmair M, Thomas C, Schmidt B (2014). Assessment of an advanced image-based technique to calculate virtual monoenergetic computed tomographic images from a dual-energy examination to improve contrast-to-noise ratio in examinations using iodinated contrast media. Invest Radiol.

[28] Wichmann JL, Varga-Szemes A, Suranyi P (2015). Transcatheter aortic valve replacement: imaging techniques for aortic root sizing. J Thorac Imaging.

[29] Khalique OK, Pulerwitz TC, Halliburton SS (2016). Practical considerations for optimizing cardiac computed tomography protocols for comprehensive acquisition prior to transcatheter aortic valve replacement. J Cardiovasc Comput Tomogr.

