# Supplementary material for: Reduction of contrast medium for transcatheter aortic valve replacement planning using a spectral detector CT: a prospective clinical trial
Source: Eur Radiol. 2023 Nov 18;34(6):4089–99. doi: 10.1007/s00330-023-10403-x (PMC11166752; doi:10.1007/s00330-023-10403-x)
Supplement: Supplementary file 1 — Supplementary file1 (DOCX 20 KB) [file 330_2023_10403_MOESM1_ESM.docx]

|  | **SNR** | | | **CNR** | | |
| --- | --- | --- | --- | --- | --- | --- |
|  | Coef. | 95% Conf. Interval | p-value | Coef. | 95% Conf. Interval | p-value |
| **Contrast medium** |  |  |  |  |  |  |
| 60ml | Ref. |  |  | Ref. |  |  |
| 50ml | -0.21 | -6.24–5.82 | 0.946 | -0.27 | -6.17–5.64 | 0.930 |
| 40ml | -1.10 | -7.23–5.04 | 0.726 | 0.00 | -6.04–6.04 | 0.999 |
| 30ml | -12.92 | -19.04– -6.81 | **<0.001** | -13.86 | -19.87– -7.85 | **<0.001** |
| **Vessel section** |  |  |  |  |  |  |
| AoAsc | Ref. |  |  | Ref. |  |  |
| AoArc | 4.04 | -0.31–8.39 | 0.069 | 3.18 | -0.70–7.07 | 0.108 |
| ThoAo | -1.68 | -6.03–2.67 | 0.448 | -0.89 | -4.78–2.99 | 0.652 |
| AbdAo | -11.65 | -16.00– -7.30 | **<0.001** | -8.49 | -12.38– -4.61 | **<0.001** |
| RCIA | -8.73 | -13.08– -4.38 | **<0.001** | -6.05 | -9.93– -2.16 | **0.002** |
| LCIA | -17.33 | -21.68– -12.98 | **<0.001** | -14.93 | -18.81– -11.05 | **<0.001** |
| REIA | -17.56 | -21.91– -13.21 | **<0.001** | -15.16 | -19.04– -11.27 | **<0.001** |
| LEIA | -16.29 | -20.64– -11.94 | **<0.001** | -14.36 | -18.25– -10.48 | **<0.001** |
| RCFA | -13.51 | -17.86– -9.16 | **<0.001** | -11.38 | -15.26– -7.49 | **<0.001** |
| LCFA | -14.55 | -18.90– -10.20 | **<0.001** | -12.75 | -16.64– -8.87 | **<0.001** |
| **Reconstructions** |  |  |  |  |  |  |
| Standard | Ref. |  |  | Ref. |  |  |
| VMI 40keV | 6.04 | 3.66–8.42 | **<0.001** | 0.71 | -1.42–2.84 | 0.513 |
| VMI 60keV | -9.80 | -12.19– -7.42 | **<0.001** | -18.43 | -20.56– -16.30 | **<0.001** |
| **Contrast medium/ Reconstructions** |  |  |  |  |  |  |
| 60ml/ Standard | Ref. |  |  | Ref. |  |  |
| 50ml/VMI 40keV | 1.09 | -2.28–4.46 | 0.525 | 1.06 | -1.95–4.07 | 0.491 |
| 50ml/VMI 60keV | 2.60 | -0.77–5.97 | 0.131 | 3.47 | 0.46–6.48 | **0.024** |
| 40ml/VMI 40keV | 0.35 | -3.02–3.72 | 0.839 | 0.61 | -2.40–3.62 | 0.691 |
| 40ml/VMI 60keV | 2.15 | -1.22–5.52 | 0.211 | 2.81 | -0.20–5.82 | 0.067 |
| 30ml/VMI 40keV | -3.33 | -6.70–0.04 | 0.053 | -2.00 | -5.01–1.01 | 0.192 |
| 30ml/VMI 60keV | 3.63 | 0.26–7.00 | **0.035** | 5.62 | 2.61–8.63 | **<0.001** |
| **Clinical Characteristics** |  |  |  |  |  |  |
| Age | 0.23 | 0.03–0.44 | **0.027** | 0.16 | -0.07–0.38 | 0.174 |
| Sex *(female)* | 2.92 | -0.19–6.03 | 0.066 | 3.20 | -0.17–6.57 | 0.063 |
| BMI | -0.88 | -1.18– -0.57 | **<0.001** | -0.83 | -1.17– -0.50 | **<0.001** |
| Agatston Score | 0.00 | -0.00–0.00 | 0.423 | 0.00 | -0.00–0.00 | 0.704 |
| Atrial Fibrillation | 2.37 | -0.80–5.55 | 0.143 | 3.40 | -0.05–6.84 | 0.053 |
| Ejection fraction *(Normal (>50%))* | Ref. |  |  |  |  |  |
| Moderately reduced (41-50%) | -0.73 | -5.34–3.89 | 0.758 | -2.33 | -7.34–2.68 | 0.361 |
| Reduced (31-40%) | -2.89 | -8.93–3.15 | 0.348 | -4.17 | -10.72–2.39 | 0.213 |
| Severely reduced (<30%) | 5.04 | -1.70–11.78 | 0.142 | 6.32 | -0.99–13.63 | 0.090 |

**S1**: Details on the mixed regression model corrected for all interaction terms and clinical characteristics as displayed in **Table 3**. *SNR, signal-to-noise ratio; CNR, contrast-to-noise ratio; BMI, body mass index; AoAsc, ascending aorta; AoArc, aortic arch; ThoAo, thoracic descending aorta; AbdAo, abdominal descending aorta; RCIA, right common iliac artery; LCIA, left common iliac artery; REIA, right external iliac artery; LEIA, left external iliac artery; RCFA, right common femoral artery; LCFA, left common femoral artery.*
